# Microbial Characteristics and Safety of Dairy Manure ComPosting for Reuse as Dairy Bedding

**DOI:** 10.3390/biology10010013

**Published:** 2020-12-28

**Authors:** Haoming Wu, Yang Wang, Lei Dong, Haiyan Hu, Lu Meng, Huimin Liu, Nan Zheng, Jiaqi Wang

**Affiliations:** 1 State Key Laboratory of Animal Nutrition, Institute of Animal Sciences, Chinese Academy of Agricultural Sciences, Beijing 100193, China; wuhaoming@caas.cn (H.W.); donglei@caas.cn (L.D.); 18894311126@163.com (H.H.); menglu@caas.cn (L.M.); liuhuiming521@163.com (H.L.); zhengnan@caas.cn (N.Z.); 2 Laboratory of Quality and Safety Risk Assessment for Dairy Products of Ministry of Agriculture and Rural Affairs, Institute of Animal Sciences, Chinese Academy of Agricultural Sciences, Beijing 100193, China; 3 Key Laboratory of Quality & Safety Control for Milk and Dairy Products of Ministry of Agriculture and Rural Affairs, Institute of Animal Sciences, Chinese Academy of Agricultural Sciences, Beijing 100193, China; 4 State Key Laboratory of Membrane Biology, Tsinghua University-Peking University Joint Center for Life Sciences, School of Life Sciences, Tsinghua University, Beijing 100084, China; wangyang881229@mail.tsinghua.edu.cn

**Keywords:** recycled manure solids, drum composter, bedding material, pathogenic bacteria, bacterial phenotype

## Abstract

**Simple Summary:**

The cost of cow manure treatment and bedding increases the operating cost of the ranch. Many ranches fill out the recycled manure solids (RMS) process to dry manure as bedding material. However, the microbial safety of RMS bedding is still uncertain, and the change of microbial diversity of the feces after each processing step is not clear. In this study, an amplified fragment sequence was utilized to analyze the microbial flora, bacterial phenotype, and metabolic function prediction of the products in the process of RMS processing. At the same time, samples of sand soil bedding material, rice husk bedding material and RMS bedding material were compared and analyzed. The results will be useful to further study the safety of RMS padding to reduce the operation cost of dairy farms and the incidence rate of mastitis.

**Abstract:**

Changes in bacterial community, phenotype, metabolic function, and pathogenic bacteria content in recycled manure solids (RMS) were analyzed by 16S rRNA sequencing, Bugbase, picrost2, and qPCR, respectively. The data from RMS bedding were compared to those of sand bedding and rice husk bedding. The results show that the proportion of potentially pathogenic bacteria among the manure flora of RMS after dry and wet separation, after composting, and after sun-cure storage was 74.00%, 26.03%, and 49.067%, respectively. Compared to RMS bedding, the proportion of potentially pathogenic microorganisms in sand bedding and rice husk bedding was higher. The picrust2 analyses show that the level of lipopolysaccharide biosynthesis changed significantly during RMS processing. In addition, the qPCR results show that composting could effectively reduce the detection and quantification of pathogens, except *Streptococcus uberis*, in RMS bedding. In general, composting is an essential step to improve the safety of bedding materials in the process of fecal treatment. However, at the same time, RMS bedding may increase the risk of mastitis caused by *Streptococcus uberis*.

## 1. Introduction

Cow manure treatment is a huge expense. At the same time, purchase of bedding material also increases the cost of farm farming [1,2]. At present, organic materials, including sawdust, wood shavings, rice husks, and rice straw, or inorganic bedding materials (e.g., sand) are used in most dairy farms. Organic materials easily lead to the colonization and growth of pathogenic bacteria [3]. Additionally, organic bedding materials must be purchased, and the cost of materials and transportation increases the cost of maintaining the ranch.

Sand is considered the most ideal bedding type for dairy cows and is strongly recommended for such purposes by livestock breeders and scientists [4]. Clean and dry sand can be used as a high-quality material to reduce the growth of bacteria related to the environment [5]. In addition, sand has good water permeability, does not easily harden, and will not cause damage to the legs and feet of dairy cows. However, when the ground of the cattle shed is cleaned, the sand bedding and cow dung are cleaned together, which cause substantial wear and damage to the equipment for handling manure, thus increasing the maintenance and repair costs to such equipment [6]. The maintenance cost of bedding material has prompted ranchers to find readily available sustainable bedding materials with high yield, and high safety, such as recycled manure solid (RMS) technology, which can dry manure for use as bedding materials [7].

Many studies have shown that the use of RMS cushion is a strategy to reduce livestock manure pollution and has many advantages [8]. After the treatment of manure, the moisture content is greatly reduced, and it becomes odorless and soft, and can be used as bedding for cows. Like sand, RMS can be placed in deep stalls. Studies have found that RMS bedding can be used to maximize the utilization of stalls, prolonging the time that dairy cows remain in bed, thereby preventing joint injury [9]. RMS materials have unique advantages: firstly, Cow manure is a type of waste that needs to be treated urgently on dairy farms. The cost to treat manure is high; however, if the manure is recycled as bedding material, the cost of purchasing such bedding material and processing cow manure is significantly reduced. Secondly, properly treated cow feces are dry and comfortable, which can improve cows’ rest, and thereby limit exercise and reduce energy consumption. More importantly, RMS bedding does not easily cause injury to cattle’s limbs and feet and can protect the health of dairy cows. Thus, an increasing number of farms are interested in the use of RMS [10,11]. The manure produced on dairy farms is properly treated by solid–liquid separation. The manure produced by dairy farms can be used as bedding materials for cattle, which can not only effectively solve problems surrounding environmental pollution in dairy farms, but also reduce the investments required for purchasing bedding materials. The surplus manure can also be used as organic fertilizer for farmlands and biogas fermentation [2,12]. Compared to sand bedding, RMS can also reduce sand mixing, reduce the difficulties associated with subsequent treatments, and reduce operation costs [7,13,14,15]. However, the use of RMS as a bedding material may also impart some risks. RMS removes moisture from the materials using physical methods such as dry and wet separation, composting, and air drying. Cows lie on bedding for substantial periods of time, and the close contact between the bedding and the nipple leads to the migration of microorganisms in the bed pad to the cow’s skin and mammary glands [16]. However, the microbial flora in RMS bedding is significantly different from that in feces. During processing, bacterial diversity in the feces of raw materials changes significantly and RMS products contain many important Gram-negative pathogens (e.g., *Klebsiella*) or food-spoiling microorganisms, such as sporulating and thermostable bacteria [15,17]. Bacterial populations, including mastitis pathogens, remain in the RMS bedding after composting, including *Klebsiella, Pseudomonas* and *Escherichia coli* [6,16]. Notably, the levels of aerobic spore formation and thermophilic bacteria in milk samples from farms using RMS are not high. RMS bedding may also increase the risk of *Streptococcus thermophilus* and *Enterococcus* in milk [6,10]. Compared with the use of sand [6] or sawdust bedding [18], the use of RMS bedding for lactating dairy cows did not increase the number of bacteria in milk [19]. However, there are still many unknowns about the biological risks associated with the use of RMS bedding for lactating dairy cows [15,20], such as the effect of RMS process on the microbial flora in feces.

The effects of RMS bedding on the microbial community in feces is unclear but is of great significance to improve the safety mechanisms surrounding fecal bedding. Due to the excellent characteristics of drum-type aerobic fermenters, including their high temperatures, uniform mixing, conduciveness to the diffusion of oxygen, and the attachment of microorganisms in the composting materials, they are effective at killing harmful microorganisms. This study used samples from the fermentation process on farms for analysis. The purpose of this study was: (1) to investigate the diversity of bacterial composition during RMS processing; (2) to analyze the diversity of bacteria in different RMS samples; (3) to compare the diversity of bacteria in sand-soil bedding, rice husk bedding, healthy cowshed bedding, and mastitis cowshed bedding; and (4) to evaluate the content of pathogenic bacteria in each RMS sample and bedding materials processed by RMS in cowsheds with healthy and mastitis cows.

## 2. Materials and Methods

### 2.1. Sample Collection and Dry Matter Determination

Sand and rice husk bedding samples were collected from dairy farms in Hebei and Heilongjiang Province in October 2019 (Hebei: sand; Heilongjiang: rice husk). In the two types of dairy farm, five bedding materials were collected and mixed into one sample, and three samples were collected with sterile sampling bags for three consecutive days. Samples for RMS processing were collected from a farm in Tianjin that used RMS bedding, including septic tank samples (RMS-1), solid–liquid separation waste liquid samples (RMS-2), solid–liquid separation samples (RMS-3), aerobic composting tank fermentation samples (RMS-4) and air-dried storage samples (RMS-5). The collected samples were immediately sealed, stored on ice, and transported to the laboratory. A total of 20 g of each sample was placed on an aluminum plate for dry matter determination. The samples were weighed wet and then placed in an oven at 100 °C for 24 h The samples were then removed from the oven and reweighed to determine the dry matter (DM) content. The remaining samples were placed at −20 °C until DNA extraction

### 2.2. DNA Extraction

A total of 200 mg of sample was weighed and transferred into a sterilized 2 mL centrifuge tube. In total, 1 mL of 70% ethanol was added to the tube, mixed, and centrifuged at 10,000 rpm for 3 min. The upper liquid fraction was then discarded. phosphate-buffered solution (PBS solution) was added to the pellet, mixed, and centrifuged at 10,000 rpm for 3 min. The upper liquid fraction was then discarded. The 2 mL tube was placed on absorbent paper for 1 min or until no liquid remained. The protocol followed that for DNA extraction provided in the E.Z.N.ATM Mag-Bind Soil DNA Kit (M5635-02, OMEGA).

### 2.3. Qualitative Analysis of the Pathogenic Bacteria Causing Mastitis

The pathogenic bacteria from mastitis cows were detected according to the bovine mastitis pathogenic bacteria nucleic acid typing detection kit (Shenzhen Bioeasy Biotechnologies Co. Ltd., Guangdong, China). TaqMan probe-based real-time PCR was used to detect common contact infectious pathogens (i.e., the NUC gene of *Staphylococcus aureus*, the CFB gene of *Streptococcus agalactiae*, the RecA gene of *Mycoplasma bovis*, the 16S rRNA gene of *Corynebacterium bovis*, the 16S rRNA gene of *Mycoplasma bovis,* the 16S rRNA gene of *Mycobacterium bovis*, and the 16S rRNA gene of *Mycoplasma* spp.) and environmental pathogens (i.e., the 16S rRNA gene of *Staphylococcus* spp., the Air gene of *Escherichia coli,* the phoe gene of *Klebsiella* spp., the 18S rRNA gene of *Prototheca* spp., the RecA gene of *Streptococcus dysgalactiae*, the RecA gene of *Streptococcus uberis*, the Plo gene of *Trueperella pyogenes*, the SSME gene of *Serratia marcescens*, the 18S rRNA gene of yeast, and the 16S rRNA gene of yeast). The qPCR amplification program was set at 50 °C for 3 min to remove contamination, followed by a pre-denaturation at 95 °C for 3 min. Following denaturation, 40 cycles of amplification were performed at 95 °C for 10 s and 60 °C for 40 s. The qPCR amplification program was then held for 3 min at 50 °C. At the end of each cycle, the fluorescence channels were set to fam, hex, Rox and Cy5 and the quantitative fluorescence data were obtained. Sample analysis was divided into the four groups, and each group used the four fluorescence quantitative analyses. In total, 16 target genes were analyzed.

### 2.4. High Throughput Sequencing

Prior to PCR amplification, a qubit2.0 DNA kit was used to quantify genomic DNA for addition to PCR. The V3-V4 universal primers 341f (5′-CCTACGGGGGGCGWGCAG-3′) and 805r (5′-GATACHVGGGTATCTATCC-3′) were used for PCR. The DNA was amplified using a Bio-Rad T 100TM thermal cycler (Hercules, CA, USA). Amplification was performed at 65 °C for 30 s, followed by 20 cycles of 94 °C for 20 s, annealing at 55 °C for 20 s, elongation at 72 °C for 30 s, and then held at 72 °C for 5 min. Illumina bridge PCR-compatible primers were introduced in the second round of PCR amplification. PCR was performed at 95 °C for 3 min after de rotation, five cycles of 94 °C for 20 s, annealing at 55 °C for 20 s, extension at 72 °C for 30 s, and finally maintained at 72 °C for 5 min. Finally, the PCR products were evaluated by 1.5% agar gel electrophoresis. The DNA was extracted and purified using the SanPrep DNA Gel Extraction Kit (SANGON Biotechnology, Shanghai, China). The Qubit2.0 DNA kit was used to accurately quantify the recovered DNA, and the final sequencing concentration was 20 pmol. The obtained materials were sequenced on the Illumina MiSeq pe300 sequencing platform (Hercules, CA, USA) at SANGON Biotechnology Co., Ltd. (Shanghai, China). Then, the data were uploaded to NCBI (PRJNA685213).

### 2.5. Bioinformatics and Statistical Analysis

During data analysis, the intergroup information of samples was masked. The forward and reverse reads from the 16S rRNA gene were imported into Qiime2-2020.02 [21]. After the data quality was confirmed, the Illumina sequence was detected and corrected using dada2 Bunge, which is an algorithm plug-in for noise reduction in qiime2 analysis, and the primers and chimeric readings were removed. Quality filtering included primer pruning and sequence truncation to remove low-quality sequences. The gene sequence was compared with the 99% greenene database to obtain the bacterial group name. A rooted phylogenetic tree was created using Miff to remove highly variable positions to reduce the noise in the tree. Fasttree-2 was used to generate a tree from the masked route. The feature table was adjusted to 12,000 sequences, thus preserving the Shannon index and Simpson index for the Evenness metric and the Observed_OTU index and Chao1 index for the diversity metric. Those indexes were calculated by qiime2′s analytical plug-in for the data of 12,000 samples described above.

The OTU abundance table without verification was used as the input, while picrust2 was used as the index. Briefly, the Rep-Seq and OTU tables were sorted after the original data were de-noised. However, it is not possible to know the specific strain information for each read, and, therefore, Picrust2 performed functional predictions based on the genetic data of samples not classified by strains. Compared with picrust1, this approach has obvious advantages in reliability, discrimination, and the breadth of prediction. Therefore, the unclassified OTU list is a prediction based on gene sequence information, rather than strain information. Combined with MetaCyc, the function of each sample was predicted. The relative predicted abundance of each functional gene was calculated. The bacterial phenotype was analyzed using Bugbase, which is a microbiome analysis tool that determines high-level phenotypes present in microbiome samples. The phenotypic contribution data, including Gram-positive, Gram-negative, biofilm-forming, potentially pathogenic, mobile-element-containing, oxygen-utilizing, and oxidative-stress-tolerant bacteria were combined with the 97% greenene annotated feature table (the data set from BugBase can only be analyzed with the results of 97% greenene) to analyze the abundance of different bacterial phenotypes [22]. The upset plot in R (version 1.2.1335) was used to analyze 1% of the bacterial families in each group [23]. Heat map analysis was performed using the Morpheus tool [24]. Prism 8 was used to analyze the relative differences in α-diversity, bacterial phenotype, bacterial family, and the functions of bacterial flora between groups. One-way ANOVA combined with the Tukey test was used to analyze the inter group differences of the α-diversity results. The corrected Q value was calculated based using the Benjamin–Hochberg procedure for False Discovery Rate(FDR) testing. Canonical Correlation Analysis (CCA) analysis was performed using pass 4.02 software.

## 3. Results and Discussion

### 3.1. Dry Mand α-Diversity

The results of the one-way ANOVA for DM and α-diversity are presented in Table 1. The moisture content of feces increased from the lower dry matter (RMS-1, 6.05 ± 0.16%) to RMS-3 (RMS-3, 20.20 ± 0.64%). The diversity and evenness index of the original feces were significantly reduced by drying, which indicated that a large number of bacteria were separated from the feces during the solid–liquid separation step, and that the remaining bacteria accounted for a large proportion of the individual strains.

Composting (RMS4) was performed using an aerobic drum-type composting machine. It was an aerobic composting system that used a horizontal drum to mix and ventilate the samples. It sped up the contact and mixing process of the solid–liquid separated cow feces and introduced oxygen through continuous rotation and accelerated the composting of cow manure in the drum. The temperature of cow feces in the drum reached 65–70 °C and the process was performed for 24–48 h. After composting, the fecal dry matter increased significantly (RMS-4, 47.52 ± 1.12%, *p* < 0.05). At the same time, compared to rms3, the diversity and evenness index of RMS-4 also increased. The Simpson index increased to 0.90 ± 0.019, indicating that composting did not only increase the diversity of bacteria in feces [2], but that the bacterial flora were more balanced.

During the process of pasture bedding management, after composting, the bedding can be used directly in the cowshed, but in many cases, the bedding material produced is more than that required in the cowshed. Therefore, the composted bedding material was usually placed in sunlight in the pasture to avoid the proliferation of bacteria. During such a process, the dry matter content decreased (RMS-5, 36.94 ± 3.53%) and the evenness index of bacteria increased, although not significantly.

The moisture content of RMS was significantly higher than that of sand and rice husk. There were more types of bacteria identified in mastitis cowshed bedding samples, healthy cowshed bedding samples, and rice husk than in sand [3,19]. In addition, compared to sand and rice husk bedding, the bacterial flora in RMS bedding was more uniform.

### 3.2. Bacterial Community Composition

**Figure 1:**
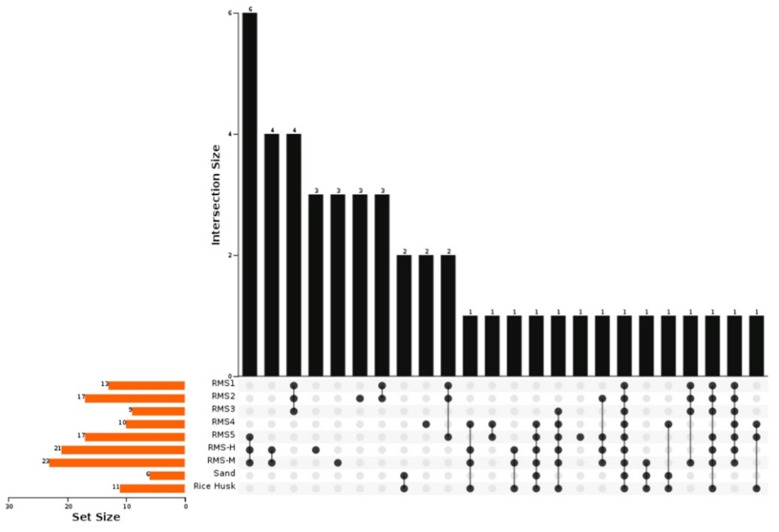
An upset plot of the core families identified in hosts. The metadata from each sample were plotted on the left bar (charts). Circles indicate samples containing accessions and the connecting bar indicates multiple overlapping samples.

In this study, a total of 394 family-level bacteria were identified, and the unclassified sequences were less than 18.88%. At the family level, there were 46 major bacterial families with at least one group of samples, with an average of more than 1%. Upset plots were used to visually analyze each group of samples [23]. It was found that the number of bacterial families in different groups was significantly different, and that RMS bedding from mastitis cowsheds (RMS-M) had the largest number of bacterial families following solid–liquid separation. The prevalence of *Ruminococcaceae*, *Bifidobacteriaceae* and *Lachnospiraceae* was very low (<1%). Moraxellaceae were abundant in all samples (proportion > 1%). The results show that the fermentation process had a great impact on the microflora of the processed feces. *Aerococcus, Carnobacteriaceae, Enterococceae* and Erysipeltrichaceae were not found in the samples following fermentation (<1%). At the same time, the relative abundances of some thermophilic bacterial families, including *Thermace* (38.92%, *Bacillaceae* (7.61%) and *Paenibacillaceae* (4.96%), became the main flora of RMS-4 samples. Thermace is often found in high-calorie environments, but rarely in milk or bedding samples. Thermace is abundant in the intestines of chronic pancreatitis (CP) mice and UC mice [25,26]. Due to the heat resistance and pathogenicity of *Thermace*, it causes adverse effects on consumers. Thus, fortunately, *Thermace* (<1%) was not found in RMS-5 samples or cowshed bedding (i.e., RMS-H and RMS-M samples) (Figure 1).

**Figure 2:**
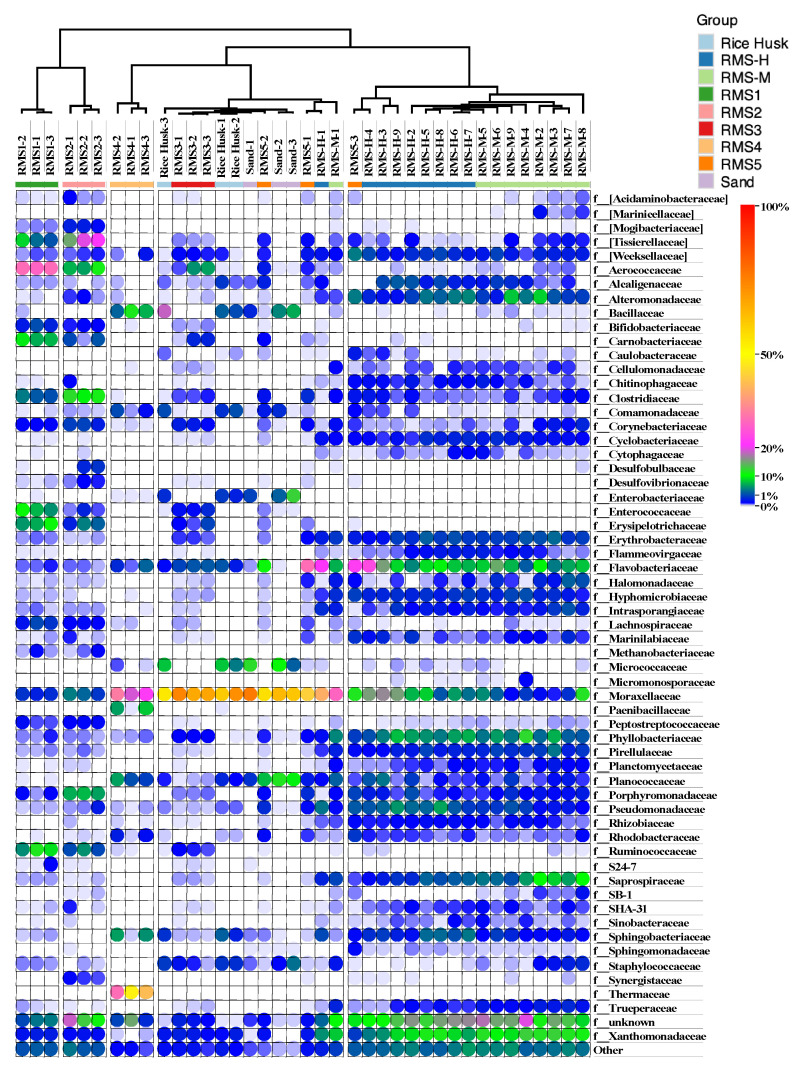
Heat map of the main bacterial families in the RMS-processing steps and different bedding samples (at least one sample with a content greater than 1%). Cluster analysis was carried out for each group using One Minus Pearson’s correlation analyses.

According to the cluster analysis results of the samples in the heatmap, the similarity between RMS bedding samples and sand and rice husk bedding samples was small (Figure 2). The bacterial diversity observed in the samples during RMS processing was also significantly different. Compared with the differences in bedding bacteria in mastitis cowsheds and healthy cowsheds, the differences of microbial flora in sand soil bedding, rice husk bedding, and RMS bedding were more significant. Dry separation bedding (RMS-3) and drying storage bedding (RMS-5) were clustered together with rice husk bedding and sand bedding. The microbial community of RMS-4 was significantly different from the four kinds of bedding samples, including rice husk, sand, RMS-M, and RMS-H samples.

There were almost no Bacillus and Enterobacteriaceae in RMS beddings [7,27]. *Micrococcaceae* and *Enterobacteriaceae* were found only in sandy soil and rice husk bedding materials. With the exception that *Bacillaceae* were found in composted samples (RMS-4, 7.61%), the content of *Bacillaceae* in bedding samples was less than 1%. It was found that the concentration of aerobic spore-forming and thermophilic bacteria in milk samples obtained from cows living on RMS bedding was not high [28]. *Bacillaceae* were found in sand (5.00%) and rice husk beddings (8.58%). The content of *Staphylococcus* in RMS-H samples (0.06%) was much lower than that in RMS-M samples (1.12%), sand (2.20%), and rice husks (2.80%).

*Moraxellaceae* was the most abundant in sandy soil (66.78%) and rice husk (62.22%), which was much higher than that of RMS-M (6.47%) and RMS-H (12.72%). *Moraxellaceae* of this Gram-negative bacterium is widely considered a pathogenic microorganism as it can cause human respiratory diseases such as asthma [29]. *M. catarhalis* is the third most common pathogen in human bacterial respiratory tract infections, including acute otitis media (AOM), sinusitis, laryngitis, and finally bronchitis or pneumonia [30]. *Moraxellaceae* was also found in milk, environmental samples, and bedding samples. *Moraxellaceae* can survive and reproduce at low temperatures, and can secrete proteases and lipases, which can cause milk protein to gelatinize and stink, shorten milk shelf life, and reduce milk quality [31].

The abundance of Pseudomonadaceae was higher in RMS-H (4.79%) and RMS-M (2.22%) samples than in sand (0.05%) and rice husk (0.56%) samples. *Pseudomonas daceae* is a psychrophilic bacterium, which can grow in large quantities at 4–10 °C. The bacteria can synthesize high-temperature-resistant lipozyme and protease, and still exhibit enzymatic activity after high temperature sterilization.

Because cows lie on their bedding for long periods of time, the contact between the mammary gland skin and the ground is substantial. Thus, this will make it easier for microorganisms in the padding to infect the breast. Bacteria on the nipple will also contaminate the milk tank during milking, thus affecting the quality of the subsequent dairy products. Therefore, the flora in environmental bedding has a great influence on milk quality. Compared with sand and rice husk bedding, the hygienic conditions of milking and milk storage from cattle living on RMS bedding should be considered.

### 3.3. Phenotype of the Bacterial Community

**Figure 3:**
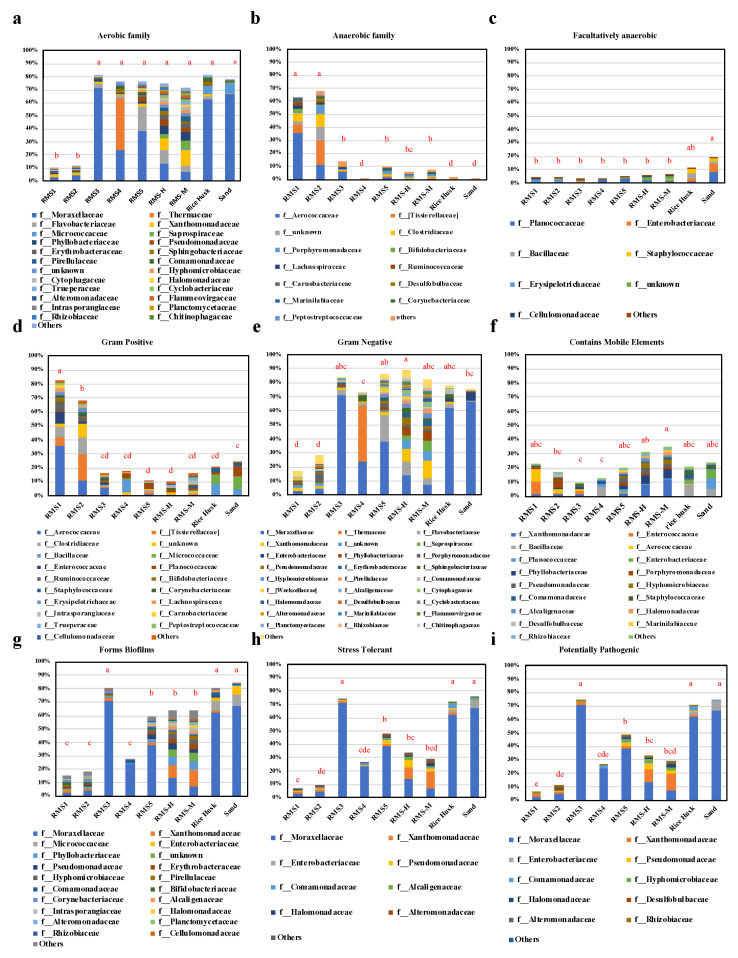
Bugbase predicted the bacterial community phenotypes in the RMS-processing steps and different bedding martials. The results of the one-way ANOVA are marked as the total proportion of samples in each group. The bacterial community phenotypes of groups (**a**–**d**) were compared (*p* < 0.05)

The functional prediction tool, Bugbase, was used to determine the abundance of potentially pathogenic bacteria. It was confirmed that rice husk bedding and sand soil bedding were carriers of pathogenic bacteria. After dry-wet separation, the relative abundance of Gram-negative bacteria increased significantly (*p* < 0.05), while the relative abundance of Gram-positive bacteria decreased significantly (*p* < 0.05). The relative abundance of potential pathogenic bacteria and biofilm formation phenotypes increased significantly (*p* < 0.05, Figure 3). Similar to the previous results, the relative abundance of Gram-negative bacteria in RMS bedding was higher than that in sandy soil and rice husk bedding [32]. It was found that the total number of Gram-negative bacteria in the used sand layer was 100–1000 times lower than that in the used RMS layer [5].

In general, this study (Figure 3) found that the relative abundance of pathogens in rms3 samples increased significantly after composting (*p* < 0.05; Figure 3i). Specifically, the relative abundance of pathogens in rms3 samples significantly increased after dry and wet separation (*p* < 0.05; Figure 3i). Additionally, the relative abundance of potentially pathogenic bacteria in different bedding materials was significantly different. The relative abundance of potentially pathogenic bacteria in sand soil and rice husk bedding was significantly higher than that in RMS bedding, and *Moraxellaceae* was the most abundant bacterial family in those samples. Therefore, our study found that both RMS, rice husk, and sand beddings provided colonization vectors for potential pathogens, although the total bacterial counts between such beddings may be different.

The formation of biofilms on bedding can be used as a protective mechanism because such microorganisms are more resistant to antibiotics and host defense mechanisms [28,33]. Therefore, bedding material is conducive to the migration of pathogens in the environment, thus increasing its potential ecological risk. There is considerable evidence that microorganisms in bedding may contaminate cow breasts and milk, and pathogenic bacteria can cause cow mastitis [16]. Bacteria form biofilms in factory pipes thus reduce the quality of dairy products [10]. In addition, the release of lipases and proteases by psychrophilic bacteria shortens the shelf life of dairy products [34,35]. During RMS processing, a large number of biofilm-forming bacteria were left in RMS-3 samples following dry and wet separation. High-temperature composting could significantly reduce the bacterial load associated with biofilms. The diversity of biofilm-forming bacteria in RMS bedding was higher than that in sandy soil and rice husk bedding, and the abundance of *Pseudomonadaceae* in RMS bedding was significantly higher than that in sandy soil and rice husk beddings. *Pseudomonadaceae* can survive and reproduce at low temperatures and thus cause milk spoilage. However, the pathogenic bacteria causing mastitis in dairy cows was higher in RMS bedding, while *Enterobacteriaceae* and *Staphylococcaceae* were only found in large quantities (>1%) in sandy soil and rice husk beddings. Therefore, as a microbial carrier, RMS bedding may not compromise milk production like sand and rice husk beddings.

### 3.4. Predicted Potential Metabolic Functions of the Bacterial Community

**Figure 4:**
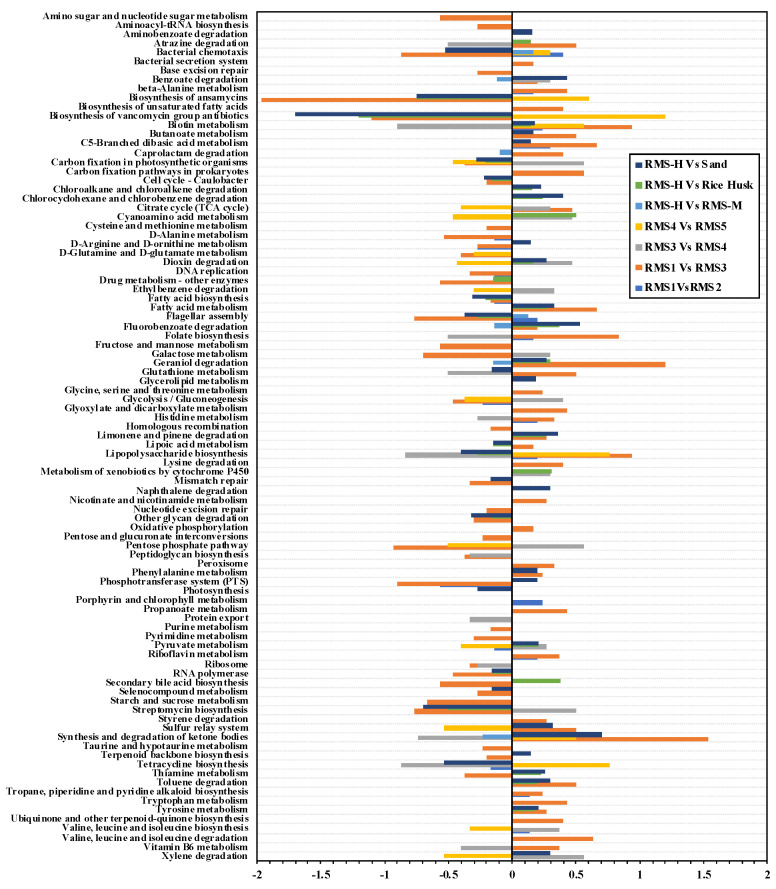
PICRUSt2 function prediction based on the MetaCyc database. One unpaired t-test per group was combined with the original False Discovery Rate (FDR)method of Benjamin and Hochberg (performed in Prism V6). Only the results with *p* < 0.05 and *q* < 0.05 were retained and displayed in the output data.

Analysis of 16S rRNA gene with the profiling phylogenetic marker genes is an important tool for the study of microbial community [36,37]. In this study, the picrust2 program was used to predict the function of dada2-filtered raw data and MetaCyc pathway pairs. A total of 185 metabolic pathways were found, of which 109 metabolic pathways were observed in >0.5% in at least one sample and accounted for >95.8% of all samples. The results of *t*-test comparisons between the processed RMS samples and the RMS-H bedding samples were compared to sand and rice husk bedding materials using pairwise comparisons (Figure 4; *p* < 0.05 and FDR < 0.05). In general, the function of the bacterial flora changed significantly after different processes. Specifically, following RMS processing, it was found that the abundance of lipopolysaccharide biosynthesis and biotin metabolism increased significantly after dry wet separation (RMS-3) and drying storage (RMS-5). Conversely, the abundance of gluconeogenesis, carbon fixation in photosynthetic organizations, and the pentose phosphate pathway increased significantly in RMS-4, and decreased in RMS-3 and RMS-5. In addition, xylene degradation, streptomycin biosynthesis, dioxin degradation, cyan amino acid metabolism, and other metabolic functions beneficial to the safety of bedding materials were significantly increased in RMS-4, thus indicating that composting manure is a good use for RMS bedding.

The abundance of bacterial chemotaxis and flagellar assembly in RMS-M was significantly higher than that in RMS-M. It should be noted that the abundance of bacterial chemotaxis and flagellar assembly in RMS-H bedding was significantly higher than that in sandy soil and rice husk. Conversely, it was significantly higher than that of the rice husk and the sand bedding. This indicated that the microbial community of RMS bedding had a significantly higher ability to synthesize antibacterial substances than sand and rice husk. It can ensure the safety of electric materials.

### 3.5. Relationships among Phenotypic Properties, Bacterial Community, and Metabolic Functions

**Figure 5:**
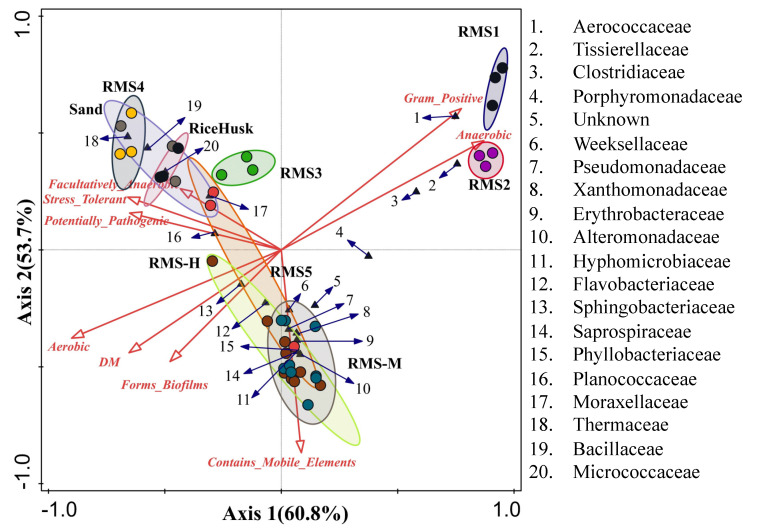
Canonical correspondence analysis of dry matter, bacterial community phenotypes, and bacterial families with proportions > 5% in the RMS processing steps and different bedding materials.

Canonical responsibility analysis further clarified the taxa associated with septic tank samples (RMS-1), liquid waste samples (RMS-2), solid–liquid separation samples (RMS-3), aerobic composting samples (RMS-4) dried storage samples (RMS-5), healthy cowshed bedding samples (RMS-H), and mastitis cowshed bedding samples (RMS-M) (Figure 5). The CCA1 and CCA2 explained 60.8% and 53.7% of the total variance, respectively. The 20 bacterial families with relative abundances higher than 5% are shown in the CCA chart. The samples of RMS-1 and RMS-2 were obviously different from the other samples. The relative abundance of *Sphingobacteriaceae* showed positive correlations with the DM, whereas the relative abundance of *Aerococcus, Tissiellaceae,* and *Clostridiaceae* showed negative correlations with the DM. RMS-3, RMS-4, and rice husk and sand bedding samples exhibited stronger correlations with potentially pathogenic bacteria, and more *Planococcaceae, Moraxellaceae*, *Thermaceae*, *Bacillaceae*, and *Micrococcaceae*.

### 3.6. Mastitis Pathogen Detection

No contact pathogens were detected in any of the RMS samples (Table 2). Only a small amount of *Klebsiella* and *Serratia marcescens* was detected in RMS bedding and RMS processing, and the highest judgment value was suspected detection (+), which had little effect on the breast health of dairy cows. Conversely, *Klebsiella* and *Serratia marcescens* were often found in fecal bedding, and yeast, Enterococcus, and Streptococcus uberis may affect the breast health of dairy cows, as well as the quality of milk and other dairy products [10]. After solid–liquid separation (RMS-3), a large volume of water in feces was separated, and *Escherichia coli* (100% suspected based on qPCR) and *Streptococcus dysgalactiae* (100% negative based on qPCR) were detected. After the feces were composted at ~70 °C (RMS-4), the detection of *Trueperella pyogenes* decreased significantly (100% negative based on qPCR) and were not detected in large amounts in subsequent samples. The survival of mastitis-causing pathogenic bacteria above 45 °C was also weak [38]. However, the detection of *Streptococcus uberis* and *Enterococcus* was significantly increased after solid–liquid separation and composting, and a suspect of those species was found in RMS bedding samples (Table 2). It has been reported that the supplementation of lab in feed can reduce the abundance of Enterococcus and Streptococcus in the mammary gland, so as to prevent mastitis [39]. Therefore, it is necessary to strengthen the prevention of *Streptococcus, enterococci* and *yeast* mastitis by using RMS bedding and adding lactic acid bacteria feed properly.

## 4. Conclusions

In general, the changes of microbial diversity and phenotypic function (i.e., the results of Bugbase analyses) in feces during RMS processing were measured. During RMS processing, the bacterial flora of fecal samples from each RMS processing step changed significantly, and the levels of stress tolerance and biofilm formation changed significantly.

In this study, the microbial composition of RMS bedding, sandy soil bedding, and rice husk bedding was compared. From such analyses, RMS bedding has a higher diversity and more types of bacteria. It was also found that there was a higher proportion of Gram-negative bacteria and potentially pathogenic bacteria in rice husk bedding and sand bedding. Thus, those findings indicate that although there are more microorganisms in RMS bedding, the threat to cow health may not be serious. Additionally, compost fermentation can effectively reduce the abundance of pathogenic bacteria in feces and the prevalence of pathogens, except *Streptococcus uberis*. Therefore, composting is an indispensable step in the process of manure treatment. However, dairy farms using RMS bedding should also be aware of the risk of mastitis caused by *Streptococcus uberis* from bedding.

## Figures and Tables

**Table 1:** One-way ANOVA of dry matter content and bacterial α-diversity of different recycled manure solids (RMS) products and different bedding materials.

	DM (%)	Diversity		Evenness	
		Observed OTU	Chao 1	Simpson	Shannon
RMS1	6.05 ± 0.16 ^d^	248 ± 14 ^ab^	250 ± 14 ^ab^	0.97 ± 0.004 ^ab^	6.33 ± 0.087 ^a^
RMS2	4.52 ± 1.81 ^d^	281 ± 6 ^a^	282 ± 6 ^a^	0.99 ± 0 ^a^	7.16 ± 0.036 ^a^
RMS3	20.20 ± 0.64 ^c^	159 ± 14 ^b^	160 ± 15 ^b^	0.61 ± 0.032 ^c^	3.34 ± 0.177 ^c^
RMS4	47.52 ± 1.12 ^a^	181 ± 32 ^ab^	184 ± 33 ^ab^	0.90 ± 0.019 ^b^	4.80 ± 0.395 ^bc^
RMS5	36.94 ± 3.53 ^b^	198 ± 31 ^ab^	199 ± 31 ^ab^	0.96 ± 0.013 ^ab^	5.91 ± 0.545 ^ab^
RMS-H	61.78 ± 3.017 ^y^	241 ± 12 ^x^	242 ± 12	0.98 ± 0.007 ^x^	6.71 ± 0.19 ^x^
RMS-M	68.75 ± 2.60 ^y^	267 ± 12 ^x^	268 ± 12	0.98 ± 0.002 ^x^	6.97 ± 0.122 ^x^
Sand	92.67 ± 1.20 ^x^	153 ± 46 ^y^	206 ± 85	0.79 ± 0.028 ^y^	3.37 ± 0.15 ^z^
Rice Husk	84.97 ± 2.43 ^x^	249 ± 16 ^x^	285 ± 18	0.84 ± 0.028 ^y^	4.44 ± 0.226 ^y^

All samples were analyzed at a depth of 12,000 reads. The abbreviations are DM: dry matter content; RMS1: septic tank sample; RMS2: waste pool sample; RMS3: sample after dry-wet separation; RMS4: sample after composting and fermentation; RMS5: sample stored in the air; RMS-H: RMS bedding sample from healthy cowshed; RMS-M: RMS bedding sample from mastitis cowshed. The bacterial α-diversity of samples from each step of processing (^a, b, c^) (*p* < 0.05) and the bacterial α-diversity of different bedding materials (^x, y, z^) were compared (*p* < 0.05).

**Table 2:** Detection of pathogenic bacteria in RMS samples by qPCR.

Target Bacteria		RMS1 (n = 3)	RMS2 (n = 3)	RMS3 (n = 3)	RMS4 (n = 3)	RMS5 (n = 3)	RMSH (n = 9)	RMS M (n = 9)
Environmental pathogens								
*Yeast (Yea)*	(+ +)		67%			67%	89%	89%
	(+)	100%	33%	100%	100%	33%	11%	11%
*Enterococcus (Ensp)*	(+ +)	100%	100%	33%	100%	100%	100%	100%
	(+)			67%				
*Klebsiella (Klsp)*	(+)	100%	100%	33%	33%	100%	100%	100%
	(−)			67%	67%			
*Escherichia coli (Ec)*	(++)	100%	67%			33%	22%	
	(+)		33%	100%	67%	67%	78%	89%
	(−)				33%			11%
*Streptococcus uberis (Sub)*	(+ + +)			67%	100%		67%	44%
	(+ +)	33%				33%	22%	11%
	(+)		67%				11%	
	(−)	67%	33%	33%		67%		44%
*Trueperella pyogenes (Tpy)*	(+ +)	100%	100%	100%				
	(+)					67%	22%	78%
	(−)				100%	33%	78%	22%
*Serratia marcescens (Sm)*	(+)		67%				11%	
	(−)	100%	33%	100%	100%	100%	89%	100%
*Streptococcus dysgalactiae (Sdy)*	(+ + +)						22%	
	(+)	67%	100%					
	(−)	33%		100%	100%	100%	78%	100%
*Protozoan (Psp)*	(−)	100%	100%	100%	100%	100%	100%	100%
Contact pathogen								
*Corynebacterium bovis (Cb)*	(−)	100%	100%	100%	100%	100%	100%	100%
*Staphylococcus aureus (Sau)*	(−)	100%	100%	100%	100%	100%	100%	100%
*Streptococcus agalactiae (Sag)*	(−)	100%	100%	100%	100%	100%	100%	100%
*Mycoplasma (Mysp)*	(−)	100%	100%	100%	100%	100%	100%	100%
*Mycoplasma bovis (Myb)*	(−)	100%	100%	100%	100%	100%	100%	100%
β-lactamase resistance gene (Lac)	Others							
	(−)	100%	100%	100%	100%	100%	100%	100%

(+ + +) strongly positive; (+ +) positive; (+) suspected positive; (−) negative. The abbreviations are DM: dry matter content; RMS1: septic tank sample; RMS2: waste pool sample; RMS3: sample after dry-wet separation; RMS4: sample after composting and fermentation; RMS5: sample stored in the air; RMS-H: RMS bedding sample from healthy cowshed; RMS-M: RMS bedding sample from mastitis cowshed.

## Data Availability

No data copyright issues.
